# Emeritus Professor Francis Kwesi Nkrumah (1935 – 2024)

**DOI:** 10.4314/gmj.v58i3.1

**Published:** 2024-09

**Authors:** Kwadwo A Koram

**Figure:**
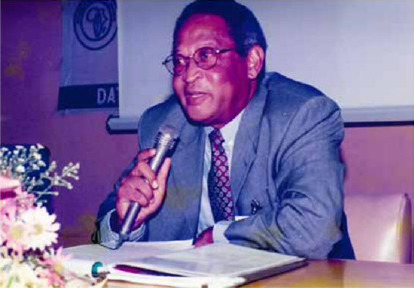
Emeritus Professor Francis Kwesi Nkrumah, former Director of the Noguchi Memorial Institute for Medical Research (1990 – 1998) and former Head of the Department of Child Health, University of Ghana Medical School (UGMS) from 1980 to 1983, died on June 30, 2024, at the age of 89

Francis Nkrumah, the eldest son of Ghana's first president, Dr Kwame Nkrumah, trained as a doctor at the University of Wurzburg and the Free University of Berlin, graduating in 1961. He returned to Ghana in 1963 and joined the Korle-Bu Teaching Hospital as a Medical Officer before going for further training in paediatrics at the Children's Hospital Medical Center, Harvard Medical School, Boston, USA, and in Public Health, receiving his MPH from Harvard School of Public Health in 1969. At the time, his father had been overthrown as head of state. Still, he advised the young Francis to return to and serve his country, a call he heeded with unwavering dedication, spurning offers from other countries, including Kenya and Tanzania, to return to Ghana.

He took up an appointment as a lecturer in the Department of Child Health at the University of Ghana Medical School. He ignored politics and concentrated on his chosen career, providing excellent teaching, research and mentorship to several students at the UGMS. He collaborated with other scientists to find a cure for Burkitt's Lymphoma, conducting several therapeutic trials in the Department of Child Health. He became the head of the Department of Child Health from 1980 – 1983.

He subsequently joined the Department of Child Health, Faculty of Medicine, University of Zimbabwe, where he was Professor and Academic Head of the Department.

In the late 1980s, Professor Akilagpa Sawyer, then Vice Chancellor of the University of Ghana, brought him back home to head the Noguchi Memorial Institute for Medical (NMIMR). Professor Nkrumah went to work with dedication and perseverance to boost the fortunes of the NMIMR and established it as the premier biomedical research institute in the country. He developed young talent and added to the physical facilities, including the provision of two Biosafety Level 3 (BSL3) labs, an experimental animals facility for pre-clinical studies, a conference facility, as well as expanding the research portfolio of the Institute. He maintained his interest in child health and global public health. He served on several international committees, including the Global Polio Eradication programme, the deployment of the meningococcal conjugate vaccine for epidemic meningitis (CSM), chairman of the Taskforce on Immunization in Africa, WHO/AFRO, member of the Governing Board of Medicines for Malaria Ventures (MMV) and a member of the Strategic Advisory Council, Children's Vaccine Programme at PATH. He was a founding member of the Paediatric Association of Ghana, a member of the International Society of Hematology, a Fellow of the Ghana Academy of Arts and Sciences, of which he became the Vice President of the Sciences section and a member of the West African College of Physicians. He served the Ghana Medical Journal in several capacities over the years, first as an editorial board member and later as a member of the International Advisory Board until his death.

Emeritus Professor Nkrumah led a life of integrity. He was humble and hardworking, with a keen sense of humour and a family man. He will be remembered as a great leader in medical education, research administration, and global health. His leadership in these fields was exemplary, earning him the respect and appreciation of all who worked with him. He will be remembered as a great leader in these fields, a legacy that will continue to inspire future generations.

He was laid to rest on 5^th^ August 2024.

May he rest in peace.

